# Electronic Communication Between Children’s Caregivers and Health Care Teams: Scoping Review on Parental Caregiver’s Perceptions and Experience

**DOI:** 10.2196/60352

**Published:** 2024-12-13

**Authors:** Mary Jo Gamper, Rebecca Singer Cohen, Maryam Esperanza Razaz, Elaina Parrillo, Clifton P Thornton, Aleksandra Wec, Kathryn McDonald, Kelly T Gleason

**Affiliations:** 1 Johns Hopkins University School of Nursing, 525 N Wolfe St, Baltimore, MD, 21205, United States, 1 (410) 955-4766; 2 Children’s Hospital of Philadelphia, Center for Pediatric Nursing Research & Evidence-Based Practice, Philadelphia, PA, United States; 3 Johns Hopkins Bloomberg School of Public Health, Baltimore, MD, United States

**Keywords:** electronic communication, patient portal, provider-patient relations, parental caregiver, relational coordination theory, patient-clinician relationship, mobile phone

## Abstract

**Background:**

Asynchronous communication via electronic modes (e-communication), including patient portals, secure messaging services, SMS text messaging, and email, is increasingly used to supplement synchronous face-to-face medical visits; however, little is known about its quality in pediatric settings.

**Objective:**

This review aimed to summarize contemporary literature on pediatric caregivers’ experiences with and perspectives of e-communication with their child’s health care team to identify how e-communication has been optimized to improve patient care.

**Methods:**

A scoping review following the Arksey and O’Malley methodological framework searched PubMed, CINAHL, Embase, and Web of Science using terms such as “Electronic Health Records” and “Communication” from 2013 to 2023 that discussed caregiver experiences and perspectives of e-communication with their child’s health care provider. Studies were excluded if they were abstracts, non-English papers, nonscientific papers, systematic reviews, or quality improvement initiatives, or pertained to synchronous telemedicine. We conducted a two-step screening process by scanning the title and abstract and reviewing the full text by two independent screeners to confirm eligibility. From an initial 903 articles identified via the database search, 23 articles fulfilled all the inclusion criteria and are included in this review.

**Results:**

Of the 23 articles meeting the inclusion criteria, 11 used quantitative methods, 7 used qualitative methods, and 5 used mixed methods. The caregiver sample sizes ranged from 51 to 3339 in the quantitative studies and 8 to 36 in the qualitative and mixed methods studies. A majority (n=17) used the patient portal that was self-categorized by the study. Secure messaging through a portal or other mobile health app was used in 26% (n=6) of the studies, while nonsecure messaging outside of the portal was used 17% (n=4) of the time and email was used 33.3% (n=8) of the time. In 19 of the studies, parents reported positive experiences with and a desire for e-communication methods.

**Conclusions:**

The literature overwhelmingly supported caregiver satisfaction with and desire for e-communication in health care, but no literature intentionally studied how to improve the quality of e-communication, which is a critical gap to address.

## Introduction

In pediatric health care, effective communication between the caregiver and clinician is pivotal for enhancing illness understanding, promoting treatment adherence, and fostering improved experiences [1-4]. Asynchronous electronic communication (e-communication), through patient portals, secure messaging, email, and SMS text messaging, is increasingly used to supplement synchronous face-to-face medical visits [5]. However, little is known about the quality of e-communication in pediatric settings.

Despite the acceleration of e-communication following the COVID-19 pandemic and the 21st Century Cures Act mandating the sharing of clinical notes with patients, there is limited evidence on the best practices, and what evidence does exist may not be applicable to the specific needs of caregivers of patients with complex needs, of children, or of both [6-10]. Limited research exists on how parental caregivers (hereinafter “caregivers”) perceive e-communication, particularly concerning its quality and perceived impact on care delivery [2,6].

The relational coordination theory is well-suited for exploring e-communication between caregivers and health care teams, as it emphasizes the quality of communication and relationships in coordinating complex, interdependent tasks such as caring for a child [11]. The relational coordination theory highlights seven key domains—shared goals; shared knowledge; mutual respect; and communication that is frequent, timely, and accurate, and aids in problem-solving—to support effective teamwork [11,12]. When applied in settings such as pediatric health care, strong relational coordination can improve communication and care outcomes [13]. Thus, incorporating relational coordination into the analysis of e-communication helps to conceptualize caregiver–health care team dynamics and inform improvements in practice.

This review was undertaken to determine contemporary literature on pediatric caregivers’ perspectives of e-communication with their child’s health care team. We aimed to (1) identify modes of caregiver–health care team e-communication; (2) assess caregiver perspectives on e-communication experiences or expectations; and (3) map findings from such studies to relational coordination domains to better understand its role in effective e-communication and how it may be leveraged to alter delivery of care systems and patient and caregiver satisfaction. The overall purpose of our study was to characterize objectives, therapeutic elements, and delivery characteristics of e-communication as a step to inform intervention development, health care practices/policies, and caregiver and health care team workflows.

## Methods

We conducted a scoping review that followed the Arksey and O’Malley [14] methodological framework and the PRISMA-ScR (Preferred Reporting Items for Systematic Reviews and Meta-Analyses Extension for Scoping Reviews) checklist (Checklist 1) [15]. We used the communication domains of the relational coordination theory to guide article synthesis [11,12].

### Study Identification

The search strategy was iteratively developed in consultation with an experienced medical librarian (Supplement 1 in Multimedia Appendix 1) and conducted in February 2023. We searched PubMed, CINAHL, Embase, and Web of Science using relevant search terms and MeSH (Medical Subject Headings) terms (Supplement 2 in Multimedia Appendix 2).

We included studies within a 10-year period that discussed caregiver experiences and perspectives of e-communication with their child’s health care provider. E-communication was defined as web-based technology that allows for asynchronous communication between a caregiver and provider, such as patient portals tethered to electronic health records (EHRs) with or without access to clinical notes, secure messaging, nonsecure messaging (eg, personal mobile, nonsecure SMS text messaging, and WhatsApp), and email. We defined the caregiver as the child’s primary caregiver and decision maker. The health care team was defined as medical professionals from various disciplines who provide comprehensive care to children, including registered nurses, nurse practitioners, physician assistants, and physicians. We limited the children’s age from 0 through 13 years due to legal, access, and privacy issues for older, adolescent patients [10,16].

Studies were excluded if they were abstracts, non-English papers, nonscientific papers, systematic reviews, or quality improvement initiatives, or if they pertained to synchronous telemedicine.

### Article Selection

We used the Covidence literature review software to conduct a two-step screening process by scanning the title and abstract and reviewing the full text by two independent screeners to confirm eligibility. The reviewers applied the inclusion and exclusion criteria to articles, discussed discrepancies as a group to reach consensus, and carefully documented decisions within the software. This method ensured a consistent and thorough review process.

### Study Synthesis

The approach to summarize and report on the findings from the identified articles varied by the research question (RQ). We first compiled articles that assessed caregiver–health care team e-communication (RQ1) by focusing on key parameters, such as the year, setting, population, and mode of e-communication [17]. We then performed a thematic analysis to synthesize information from articles that reported on caregiver satisfaction related to e-communication (RQ2) and the seven relational coordination domains (RQ3). The analysis pertaining to RQ2 and RQ3 involved mapping the findings of the mode of e-communication onto the relational coordination domains, under the hypothesis that the higher the number of relational coordination domains identified per article, the higher the caregiver satisfaction [12].

## Results

### Overview

**Figure 1.**
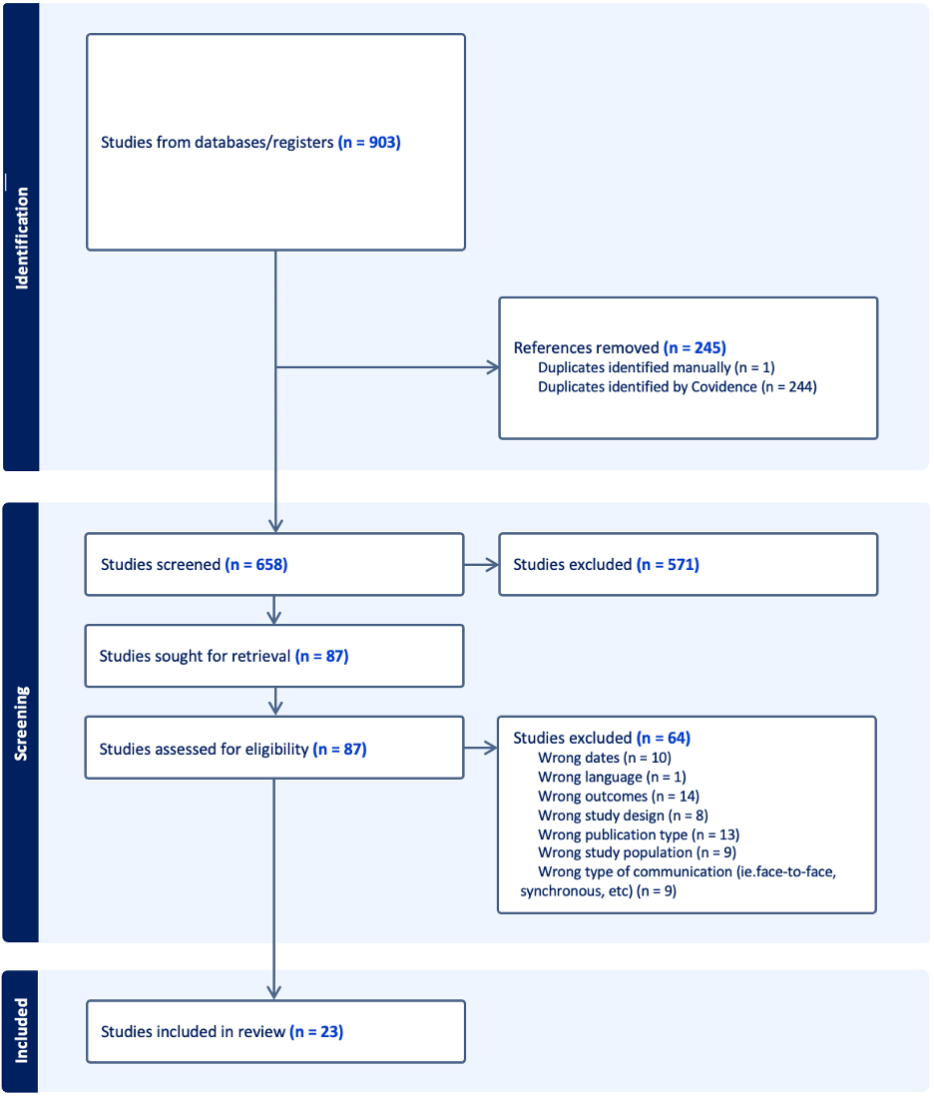
PRISMA (Preferred Reporting Items and Systematic Reviews) flow diagram showing the literature search and study inclusion.

In total, 903 articles were identified via the database search. After the duplicates were removed, 658 articles remained for title and abstract screening. Eighty-seven percent (571/659) of the articles were excluded, resulting in 87 full-text studies assessed for eligibility. During the full-text review, 74% (64/87) of the articles were excluded, which resulted in 23 articles included in this review. Figure 1 shows the study selection process in a PRISMA diagram [15].

### Basic Characteristics of the Included Studies

The characteristics of the included articles are summarized in Table 1 and individually presented in Supplement 1 in Multimedia Appendix 1. Of the 23 articles included in this review, 11 used quantitative methods, 7 used qualitative methods, and 5 used mixed methods. Sixteen studies (70%) were conducted in the United States, and one-third (n=8, 34.7%) were published after 2020. Half of the studies were conducted in an ambulatory setting (n=13, 56.5%), with the next most frequent location being an inpatient setting (n=8, 34.7%), of which 2 (8%) were in the neonatal intensive care unit. Only 4 (17.4%) were conducted in the community setting. One-third (n=7, 30.4%) of the studies involved populations with chronic illness or complex medical needs. Half (n=12, 52.2%) of the studies included participants in addition to caregivers, such as health care providers, adult patients, and teachers. The caregiver sample sizes ranged from 51 to 3339 in the quantitative studies and 8 to 36 in the qualitative and mixed methods studies. Over half of the 11 caregiver-only studies included 80% or greater female participants.

**Table 1.** Basic characteristics of the included studies.^a^

	Overall (N=23), n (%)		EHR^b^/patient portal (n=17), n (%)		Other^c^ (n=13), n (%)	
**Year**						
2013‐2016	8 (34.8)		6 (75.0)		5 (62.5)	
2017‐2020	7 (30.4)		5 (71.4)		4 (57.1)	
2021‐2023	8 (34.8)		6 (75.0)		4 (50.0)	
**Study Design**						
Quantitative	11 (47.8)		8 (72.7)		6 (54.5)	
Qualitative	7 (30.4)		6 (85.7)		3 (42.9)	
Mixed methods	5 (21.7)		3 (60.0)		4 (80.0)	
**Participants**						
Parents	23 (100.0)		17 (73.9)		13 (56.5)	
Health care worker	9 (39.1)		5 (55.6)		6 (66.7)	
Adult patient	1 (4.3)		1 (100.0)		0 (0.0)	
Teacher	1 (4.3)		0 (0.0)		1 (100.0)	
**Setting**						
Ambulatory	13 (56.5)		11 (84.6)		7 (53.8)	
Inpatient/ICU^d^	8 (34.8)		7 (87.5)		4 (50.0)	
Community-based	4 (17.4)		2 (50.0)		4 (100.0)	
Web-based	1 (4.3)		0 (0.0)		1 (100.0)	
**Mode of e-communication**						
EHR/patient portal	17 (73.9)		—^e^		—	
Secure messaging	6 (26.1)		—		—	
Email	8 (34.7)		—		—	
Nonsecure SMS text messaging	4 (17.4)		—		—	
Phone	3 (13.0)		—		—	
Other	2 (9.7)		—		—	

a Individual studies may have more than one category of participants, setting, or mode of e-communication, so the overall percentage corresponds to 23 studies. The columns EHR/patient portal and Other (any mode that is not EHR/patient portal as described by a study) may add up to more than the number of overall studies in a row because studies may include more than one mode of e-communication. The percentage in those columns is the number of studies/number of overall studies in the corresponding row.

b EHR: electronic health record.

c Other includes secure messaging (n=6), email (n=8), SMS messaging (n=4), phone (n=3), fax (n=1), and web chat (n=1).

d ICU: intensive care unit.

e Not applicable.

### Modes of Communication

The studies were assessed for the mode of e-communication utilized for caregiver–health care team communication and may include more than one mode (Table 2; Supplement 3 in Multimedia Appendix 3). A majority (n=17) used the patient portal that was self-categorized by the study. Secure messaging through a portal or other mobile health app was used in 26% (n=6) of the studies, while nonsecure messaging outside of the portal was used 17% (n=4) of the time and email was used 33% (n=8) of the time. Health care team phone messaging services and facsimiles were cited at 13% (n=3) and 4% (n=1), respectively. Notably, secure messaging was not described in studies prior to 2016; however, patient portals and email were actively referenced throughout the entire study period and could have included secure messaging. Correspondingly, SMS text messaging did not appear in studies after 2021.

**Table 2.** Evidence of relational coordination theory concepts in the literature.

Author, year	Mode of communication						7 domains of relational coordination						
	EHR^a^/portal	Secure messaging	Email	SMS text messaging	Phone	Other	Relationships with			Communication with			
							Shared goals (n=11)	Shared knowledge (n=16)	Mutual respect (n=10)	Frequent (n=11)	Timely (n=20)	Accurate (n=14)	Problem-solving (n=6)
Britto et al [18], 2013	✓						✓	✓	✓	✓	✓	✓	✓
Clark and Pledge [19], 2015	✓												
Fiks et al [20], 2015	✓						✓	✓			✓	✓	
Aldekhyyel et al [21], 2018	✓										✓	✓	
Kelly et al [22], 2019	✓						✓	✓	✓	✓	✓	✓	✓
Amirav et al [23], 2020	✓							✓				✓	
Bell et al [24], 2021	✓						✓	✓	✓	✓	✓	✓	
Kelly et al [25], 2021	✓						✓	✓	✓	✓	✓	✓	
Sarabu et al [26], 2021	✓							✓	✓		✓	✓	
Smith et al [27], 2022	✓						✓	✓	✓	✓	✓	✓	✓
King et al [28], 2017	✓	✓						✓			✓		
Kelly et al [29], 2023	✓	✓					✓	✓	✓	✓	✓	✓	
Weatherly et al [30], 2019	✓	✓			✓						✓	✓	
Nadia et al [31], 2022	✓	✓	✓								✓		
Weems et al [32], 2016	✓	✓	✓	✓			✓	✓		✓	✓		
Dudas and Crocetti [33], 2013	✓		✓	✓							✓		
Horsky et al [34], 2014	✓		✓		✓	✓	✓	✓			✓	✓	✓
Parpia et al [35], 2021		✓	✓		✓		✓	✓	✓	✓	✓		✓
Schiller et al [36], 2013			✓				✓	✓	✓	✓	✓	✓	✓
deJong et al [37], 2017			✓								✓		
Adams et al [38], 2021			✓	✓				✓	✓	✓	✓		
Globus et al [39], 2016				✓				✓		✓		✓	
Kaskinen et al [40], 2018						✓					✓		

a EHR: electronic health record.

### Caregiver Experiences and Expectations for E-Communication in Pediatric Health Care

Overall, when studies are evaluated for positive versus negative caregiver experiences with e-communication use, a majority (n=20, 87%) identified positive experiences, with 3 studies finding more negative impressions of e-communication primarily due to cumbersome electronic system interface, not specifically the quality of communication [30,34,38].

Caregiver utilization of e-communication methods varied widely across studies, but common themes emerged. Particularly, 18 studies highlighted caregiver satisfaction through enhanced access to information, faster communication, and increased preparedness. For example, the ability to access patient information through portals, as noted in both national surveys [19] and qualitative studies [22], empowers caregivers by providing them with immediate, transparent access to their child’s health data. The capacity to view laboratory and clinical data, request referrals, or schedule appointments through such systems reduces logistical barriers, offering convenience and a sense of control [19]. Moreover, electronic communication tools, such as portal messaging, email, and texting, reduce caregivers’ anxiety by ensuring quicker, more precise interactions with health care teams. For example, mothers in neonatal intensive care unit settings were able to better understand their infant’s condition and prepare for postdischarge care through improved clarity and communication speed [41]. However, even as e-communication platforms demonstrate significant benefits, nearly half of caregivers still prefer face-to-face interactions [41]. This suggests that while many caregivers value face-to-face interactions for nuanced discussions, using e-communication in conjunction helps improve access, alleviate burdens, and better equip caregivers for comprehensive conversations, contributing to overall satisfaction [22].

Other studies highlight the importance of a dynamic, two-way exchange of information in e-communication systems. For instance, a qualitative study of parents of children with chronic illnesses found that a secure messaging portal reduced barriers to getting timely answers, which in turn fostered feelings of control, independence, and reassurance [18]. A randomized controlled trial study using the MyAsthma portal demonstrated how ongoing bidirectional communication between families and clinicians supports shared decision-making by enabling real-time adjustments to treatment plans [20]. A cross-sectional survey further revealed that parents are not passive recipients but active participants, frequently initiating communication through messages or requests, emphasizing their active role in managing their child’s health care [42].

Three studies focused on the caregivers’ perceptions of reading their child’s electronic progress notes [24-26]. One survey found that access to their child’s clinical progress note positively impacted the caregivers’ level of “confidence and trust” in their health care and enabled caregivers to feel like a “part of that team.” [24] In qualitative focus groups, an inpatient-facing study found that caregivers believed that communication via access to clinical notes would “enhance the partnership and collaboration” between caregivers and the health care team and support higher standards for communication accuracy and accountability [25]. A survey on the impact of clinical notes being made available online to patients on the patient-physician relationship demonstrated that a parent’s perception of their child’s physician was generally positive with access to notes and 15% of parents used the ability to contact their provider about something they read in the note [26].

Three studies focused on the impact and satisfaction that SMS text messaging has on parental caregivers. An intervention focused on sending updates to parents via an SMS text regarding clinical updates significantly improved parental satisfaction with the medical treatment, the information provided, and the communication with their neonatal intensive care unit–admitted infants’ medical staff. Parents also indicated perceived improvements in medical staff’s availability, patience, approachability, and trust after the intervention. In a recent mixed methods study evaluating the use of a non–EHR-tethered secure messaging system compared to emails and phone calls, caregivers enjoyed the “laid-back, casual quick messages” of SMS text messaging; however, they felt email was the most convenient method because they were already logged into their email and they could “communicate with multiple providers at the same time” [35]. In a study involving an intervention of web-based chat consultation with resident physicians, caregivers felt their concerns and questions were “well handled” by the extra time with providers via the web chat, despite it not being face-to-face [40].

Two additional studies focused on attitudes specifically toward emailing the child’s health care team. A majority (n=178, 78%) of caregivers from a 2013 cross-sectional study in an urban pediatric primary care clinic showed interest in communicating with their child’s providers by this method and attitudes were favorable, with three-quarters of email users reporting that it would improve communication with their provider [33]. A mixed methods study that elicited parents’ perspectives on this topic for informing medical student training found that most participants placed high value on a provider’s “ability to communicate, respectfully, and empathetically in email.” [36]

For the 3 studies that discussed the needs of parents of children with complex needs, the modes of e-communication involved patient portal, secure messaging, email, and SMS text messaging, which were universally believed to enhance the patient/caregiver and health care team relationship [34,35,38]. However, a theme of operational barriers emerged from each study. For example, a lack of integration and interoperability of e-communication systems within and across institutions and professions was found to add considerable effort to both the caregivers and the health care team [34].

### Evidence of Relational Coordination Concepts

Of the 7 concepts of the relational coordination theory, “timely” e-communication was identified as a key characteristic in a majority of the studies (n=20) [43], followed by the relationships with “shared knowledge” (n=16; Table 2). Thematic analysis noted “accurate” communication in approximately two-thirds of the studies (n=14), and both “frequent” communication and relationships with “shared goals” were associated with half of the studies (n=11). The relationship with “mutual respect” was detected in fewer than half of the studies (n=10), and “problem-solving” communication was only identified in one-quarter of the studies (n=6). Four references, each quantitative in design, were only coded for the “timely” relational coordination domain [31,33,37,40]. One reference did not demonstrate evidence of relational coordination concepts [19]. These counts help map the prevalence of each concept in the literature and provide insight into the degree to which relational coordination principles have been explored in the context of caregivers’ experiences.

## Discussion

### Key Findings

This scoping review included 23 articles and identified a small body of literature from the last decade focusing on parental caregiver’s perspectives on e-communication with their child’s health care team. Overwhelmingly parents reported positive experiences with and a desire for e-communication methods. This is particularly relevant to the times as the 21st Century Cures Act [9] has increased the sharing of clinical notes and test results asynchronously with patients/caregivers. However, there is sparse data on how parents/caregivers perceive such new platforms of communication, given that only 5 out of 23 studies include analysis of access to clinical notes. Overall, the findings suggest positive caregiver experiences with e-communication. Negative perceptions were largely due to technological barriers, including workflow disruptions and underdeveloped communication platforms. These barriers impacted the ease of communication but were not explicitly linked to the quality of the interaction between caregivers and health care team.

The literature spans care settings and illness acuity, and includes chronic, primary, and specialty care. Female caregivers were the most prevalent among the caregiver populations studied. Most studies examined lived experiences of participants, while a small number addressed anticipated expectations [25,32,36]. Although most studies had aims centered around the theme of caregiver perceptions of communication via various modes of e-communication with their child’s health care team, the lack of standardized measures for the quality of e-communication made systematically looking across studies for factors to improve the caregiver perception difficult.

### Gaps in the Literature

Despite the well-documented growing use of e-communication between patients/caregivers and the health care team, little is known about the characteristics and quality of the conversations that occur and whether the quality of the conversations impacts outcomes [44,45]. In rare instances where the content of e-communication has been analyzed, differences have been identified compared to in-person communication. For example, one study of e-communication via online portals at a large medical center showed a reduction in partnership-building language and supportive talk compared to in-person conversations [46]. Although this study did not measure the effects of this shift, it is possible that such changes could negatively impact the perceived quality of communication. Since evidence exists within nonelectronic communication (ie, face-to-face communication) about how specific content affects perceived quality [6], it is important to determine if those same characteristics and quality preferences apply to e-communication.

Although different modes of communication were studied, trends in preference for mode of e-communication may have changed over time due to increased use of and advances in EHRs and patient portals in the last several years [47]. However, none of the studies necessarily explored whether EHRs and patient portals were the primary modes of communication being routinely utilized. For example, a study may have specifically been asking about patient portals, but the caregiver and health care team may actually communicate most often via nonencrypted texts. SMS text messaging a health care team member’s personal cell phone, as opposed to using a secure messaging service through a portal or web-based application, was not evident in this literature search; however, the practice is commonplace [48].

### Relational Coordination Theory and Its Relevance

The relational coordination theory emphasizes the importance of shared goals, shared knowledge, and mutual respect in communication. Although the 7 key domains of the theory are present in part in a majority of the studies, both quantitative and qualitative, no study explicitly used the relational coordination theory as a framework to aid in conceptualization, measures of communication, or design of tools or interventions to improve e-communication. However, given the theory’s focus on the quality of relationships through communication between team members, it is highly relevant for understanding and improving e-communication between caregivers and health care teams. To strengthen the application of the relational coordination theory, future studies should integrate it into the analysis of caregiver-provider e-communication to better assess how shared goals and mutual respect are fostered or hindered through electronic means, and how these domains affect the quality of care.

### Limitations of the Literature

There are limitations to our ability to generalize the findings of this review to the national landscape of patient portals, as the majority of studies were conducted at academic health care institutes. This introduces a potential bias in the sample, as academic health care institutes may have different patient populations, resources to support an EHR/portal, and communication practices compared to other types of health care settings (eg, community hospitals and private practices). There was an absence in the literature on the use of nonsecure SMS text messaging despite its widespread application. Furthermore, not restricting the studies by country enhances the diversity in the review, health care, communication, and e-communication practices that vary across countries, which may also affect generalizability.

Populations in this scoping review were not diverse in sex, with possible underrepresentation of male caregivers in a majority of the studies. The rationale for this sex difference in contemporary research was not explained in any of the studies but likely represented convenience sampling. However, fathers, particularly fathers from disadvantaged backgrounds, have historically been underrepresented in pediatric research, and their lack of recruitment may impede our understanding of paternal effects on children’s health and the development of effective family interventions [49].

The participants were not diverse in origins, cultures, or native languages, which appears to be due to language-based inclusion/exclusion criteria cited in some studies, as well as racial and ethnic disparities in technology needed for patient portal offers, access, and use [50]. Workarounds to language barriers might include the use of automated translation software, such as Google Translate, by caregivers or providers to attempt to facilitate asynchronous e-communication; however, the translation is not accurate across languages and only contributes to worsening of health inequity [51,52]. As the perspectives identified in the studies reflect English language speakers, caregivers from other linguistic or cultural backgrounds might have different expectations or priorities for e-communication.

Finally, the lack of representation of the independent child voice in the review is a significant limitation. By primarily focusing on caregivers, the review may not fully capture the perspectives and preferences of the primary participant in pediatric care—the patients themselves who may have high digital literacy. Measurement of children’s views in communication, particularly in adolescents and young adults, and their impact on outcomes and experiences is crucial for a comprehensive understanding of pediatric e-communication dynamics.

### Conclusions

This review provides a foundation for understanding the evidence base regarding how e-communication may be used to drive improved patient outcomes, experiences, or health care system workflows. The gap in the literature regarding pediatric caregiver and health care team e-communication, specifically the lack of evaluation of the quality of the communication from the caregiver perspective, is urgent to address given the rapid proliferation of e-communication in health care [47]. Investigating this critical void can contribute valuable insights to health care policy and practice guidelines. As e-communication tools become increasingly integrated into health care delivery, understanding the needs, preferences, and experiences of caregivers is essential for optimizing communication quality and enhancing experiences.

## Supplementary material

10.2196/60352 Multimedia Appendix 1 Keyword search and strategy.

10.2196/60352 Multimedia Appendix 2 Mode of electronic communication sorted chronologically by year of publication.

10.2196/60352 Multimedia Appendix 3 Studies on electronic communication between parental caregivers and their child’s health care providers.

10.2196/60352 Checklist 1 PRISMA-ScR (Preferred Reporting Items for Systematic Reviews and Meta-Analyses Extension for Scoping Reviews) checklist.

## Abbreviations

EHR: electronic health record
MeSH: Medical Subject Headings
PRISMA-ScR: Preferred Reporting Items for Systematic Reviews and Meta-Analyses Extension for Scoping Reviews
