# Supplementary material for: Electronic Communication Between Children’s Caregivers and Health Care Teams: Scoping Review on Parental Caregiver’s Perceptions and Experience
Source: JMIR Pediatr Parent. 2024 Dec 13;7:e60352. doi: 10.2196/60352 (PMC11661689; doi:10.2196/60352)
Supplement: Multimedia Appendix 1 [file pediatrics-v7-e60352-s001.docx]

**Supplement 2. Keyword search and strategy.**

| **PubMed**  128 results |
| --- |
| "Electronic Health Records"[Mesh] OR "Clinical notes" [tiab:~2] OR "Clinical note" [tiab:~2] OR "Open notes" [tiab:~2] OR "Open Note" [tiab:~2] OR "Physician notes" [tiab:~2] OR "Patient portal" [tiab:~2] OR "Electronic health record" [tiab] OR "EHR" [tiab] OR "EMR" [tiab] OR "electronic medical record" [tiab:~2] OR "Patient portals" [Mesh] OR "electronic mail" [Mesh] OR "electronic mail" [tiab:~2] OR "e-mail" [tiab]  AND  "Communication"[Mesh] OR "Communicat*" [tiab]  AND  "Physician-Patient Relations"[Mesh] OR "Professional-Patient Relations"[Mesh] OR "Physician patient" [tiab:~2] OR "doctor patient" [tiab:~2] OR "nurse patient" [tiab:~2] OR "provider patient" [tiab:~2] OR "physicians" [Mesh] OR "nurses" [Mesh] or "physician*" [tiab] OR "nurse*" [tiab] OR "provider" [tiab]  AND  "Parents"[Mesh] OR "parent*" [tiab] OR "father*" [tiab] OR "mother*" [tiab] OR "patients" [Mesh] OR "patient*" [tiab] |
| **Embase**  462 results |
| 'electronic health record'/exp OR 'e-mail'/exp OR 'patient portal'/exp OR (((clinical OR physician OR open) NEAR/2 (note OR notes)):ti,ab,kw) OR 'electronic medical records':ti,ab,kw OR email:ti,ab,kw OR 'electronic health record':ti,ab,kw OR emr:ti,ab,kw OR ehr:ti,ab,kw OR ((patient NEAR/2 portal*):ti,ab,kw)) AND ('interpersonal communication'/exp OR communicat*:ti,ab,kw) AND ('professional-patient relationship'/exp OR 'physician'/exp OR 'nurse'/exp OR (((physician* OR provider* OR nurse* OR doctor*) NEAR/2 patient*):ti,ab,kw) OR physician*:ti,ab,kw OR nurse*:ti,ab,kw OR provider*:ti,ab,kw) AND ('parent'/exp OR father*:ti,ab,kw OR mother*:ti,ab,kw OR parent*:ti,ab,kw) |
| **CINAHL**  156 results |
| ( (MH "Electronic HealthRecords+") OR (MH"Email") OR (MH "PatientPortals") ) OR TI (((clinical OR physician ORopen) N2 (note OR notes))OR electronic medicalrecords OR email ORelectronic health record OREMR OR EHR OR (patientN2 portal*) ) OR AB (((clinical OR physician OR open) N2 (note OR notes))OR electronic medical records OR email ORelectronic health record OREMR OR EHR OR (patientN2 portal*) )  AND  (MH "Communication+")OR communicat*  AND  ( (MH "Physician-PatientRelations") OR (MH"Professional-PatientRelations+") OR (MH"Nurse-Patient Relations")OR (MH "Physicians+")OR (MH "Nurses+") ) ORTI ( ((physician* ORprovider* OR nurse* ORdoctor*) N2 patient*) ORphysician* OR nurse* ORprovider* ) OR AB (((physician* OR provider*OR nurse* OR doctor*) N2patient*) OR physician*OR nurse* OR provider* )  AND  (MH "Parents+") OR TI (father* OR mother* ORparent* ) OR AB ( father*OR mother* OR parent* ) |
| **Web of Science**  156 results |
| (((TS=(“electronic health record” OR “e-mail” OR “patient portal” OR ((clinical OR physician OR open) NEAR/2 (note OR notes)) OR “electronic medical records” OR email OR “electronic health record” OR emr OR ehr OR ((patient NEAR/2 portal*)))) AND TS=((“interpersonal communication” OR communicat* ))) AND TS=((“professional-patient relationship” OR “physician” OR “nurse” OR ((physician* OR provider* OR nurse* OR doctor*) NEAR/2 patient*)) OR physician* OR nurse* OR provider*)) AND TS=((“parent” OR father* OR mother* OR parent*)) |
