# Supplementary material for: Electronic Communication Between Children’s Caregivers and Health Care Teams: Scoping Review on Parental Caregiver’s Perceptions and Experience
Source: JMIR Pediatr Parent. 2024 Dec 13;7:e60352. doi: 10.2196/60352 (PMC11661689; doi:10.2196/60352)
Supplement: Multimedia Appendix 2 [file pediatrics-v7-e60352-s002.docx]

**Supplement 3. Mode of electronic communication, sorted chronologically by year of publication.**

| **Author** | **Year** | **EHR / Patient portal n=17** | **Secure messaging n=6** | **Email  n=8** | **Text messaging n=4** | **Phone  n=3** | **Other  n=2** |
| --- | --- | --- | --- | --- | --- | --- | --- |
| Britto | 2013 | X |  |  |  |  |  |
| Dudas | 2013 | X |  | X | X |  |  |
| Schiller | 2013 |  |  | X |  |  |  |
| Horsky | 2014 | X |  | X |  | X | X (Fax) |
| Clark | 2015 | X |  |  |  |  |  |
| Fiks | 2015 | X |  |  |  |  |  |
| Globus | 2016 |  |  |  | X |  |  |
| Weems | 2016 | X | X | X | X |  |  |
| deJong | 2017 |  |  | X |  |  |  |
| King | 2017 | X | X |  |  |  |  |
| Aldekhyyel | 2018 | X |  |  |  |  |  |
| Kaskinen | 2018 |  |  |  |  |  | X (Web chat) |
| Kelly | 2019 | X |  |  |  |  |  |
| Weatherly | 2019 | X | X |  |  | X |  |
| Amirav | 2020 | X |  |  |  |  |  |
| Adams | 2021 |  |  | X | X |  |  |
| Bell | 2021 | X |  |  |  |  |  |
| Kelly | 2021 | X |  |  |  |  |  |
| Parpia | 2021 |  | X | X |  | X |  |
| Sarabu | 2021 | X |  |  |  |  |  |
| Nadia | 2022 | X | X | X |  |  |  |
| Smith | 2022 | X |  |  |  |  |  |
| Kelly | 2023 | X | X |  |  |  |  |
