# Supplementary material for: Electronic Communication Between Children’s Caregivers and Health Care Teams: Scoping Review on Parental Caregiver’s Perceptions and Experience
Source: JMIR Pediatr Parent. 2024 Dec 13;7:e60352. doi: 10.2196/60352 (PMC11661689; doi:10.2196/60352)
Supplement: Multimedia Appendix 3 [file pediatrics-v7-e60352-s003.docx]

**Supplement 1. Studies on electronic communication between parental caregivers and their child's health care providers.**

| **Author** | **Year** | **Title** | **Study Design** | **Study Aim** | **Study Participants** | **Setting** | **Institution** | **Duration** | **Main Findings** |
| --- | --- | --- | --- | --- | --- | --- | --- | --- | --- |
| Amirav, I [48] | 2020 | An Asthma Specialist's Consult Letter: What Do Parents Think about Receiving a Copy? | Quantitative | To examine parents’ perceptions about receiving a hard copy EMR-generated letter from a pediatric pulmonologist at the end of their child’s first asthma consult visit. | Parents of children (n=51) | Ambulatory | Not disclosed | 12 months | Parents value and want to receive a hard copy of their child’s asthma specialist’sEMR-generated summary letter at the end of the visit, and they would like to receive information of this nature from other clinicians |
| Bell, S [29] | 2021 | Tackling Ambulatory Safety Risks Through Patient Engagement: What 10,000 Patients and Families Say About Safety-Related Knowledge, Behaviors, and Attitudes After Reading Visit Notes | Quantitative | Systematically examine the effect of reading notes on patient and family perceptions of safety-related knowledge, behaviors, and attitudes | Parents/guardians of pediatric patients (n=3339);  Adult patients (n=6913) | Ambulatory (1 adult, 1 pediatric) | Not disclosed | 4 months | Transparent visit notes can engage patients and families in at least some areas of ambulatory risk |
| Clark, S [49] | 2015 | A National Survey of Parent Perspectives on Use of Patient Portals for Their Children's Health Care | Quantitative | To assess parents' current utilization and future willingness to use patient portals to conduct business and patient care activities for their child | Parents of children 0-17 years (n=1420) | Ambulatory | National survey | Not disclosed | Patient portals are not currently a significant mode of parent-provider interactions in the pediatric setting |
| deJong, N [35] | 2017 | Enhanced Access and Parents' Preferred Contact fora Child's Chronic Condition | Quantitative | To assess whether the perception of enhanced access by parents in their child's primary care and main specialty practices is associated with preference for contacting either practice when problems arise with a child's chronic condition | Parents whose children <18 years use both primary and specialty practices (n=609) | Ambulatory | Tertiary-care children's hospital in southeastern US | 2 months | We found that a majority of parents would prefer to contact a specialist with concerns about a child's chronic condition exacerbation and that preference was related to perception of enhanced access at the child's main specialist practice |
| Dudas, R [32] | 2013 | Pediatric Caregiver Attitudes Toward Email Communication: Survey in an Urban Primary Care Setting | Quantitative | To document pediatric caregiver attitudes toward and access to these technologies in an urban pediatric primary care clinic | Pediatric caregiver-Child aged 0-21 year dyads (n=229) | Ambulatory | Johns Hopkins University | 14 months | Most caregivers in our urban population have access to email and are interested in communicating with their child's providers by this method, although only 11% currently communicate with their provider via email. Overall, attitudes toward email were favorable with three-quarters of email users reporting that email would improve communication with their provider and be satisfying |
| Fiks, A [25] | 2015 | Parent-Reported Outcomes of a Shared Decision-Making Portal in Asthma: A Practice-Based RCT | Quantitative | Test the feasibility, acceptability, and impact of an innovative, EHR-linked patient portal with decision support directed at both families and clinicians on asthma outcomes | Parents/legal guardians of children aged 6-12 years (n=60) | Ambulatory (1 urban, 1 suburban) | The Children's Hospital of Philadelphia | 12 months | Use of an EHR-linked asthma portal was feasible and acceptable to families and improved clinically meaningful outcomes |
| Globus, O [50] | 2016 | The use of short message services (SMS) to provide medical updating to parents in the NICU | Quantitative | To evaluate the impact of using SMS technology for updating parents of preterm infants with medical information on parents and nursing staff to increase parent's satisfaction and parent-staff communication | Parents of infants hospitalized in the NICU (n=91); Nurses (n=47) | NICU | Sheba Medical Center | Not disclosed | SMS updating is an easy and user-friendly technology that enriches the modalities of information delivery to parents of hospitalized preterm infants |
| Kaskinen, A [31] | 2018 | Pediatric Web-Based Chat Services for Caregivers of Children: Descriptive Study | Quantitative | To describe chat conversations between caregivers and physicians in a Web-based chat service to determine the factors that should be considered when planning a similar chat service. To evaluate whether caregivers considered the consultations helpful, whether physicians considered they could answer caregivers' questions, and whether further face-to-face medical contact was needed | Caregivers of children (n=98) | Web-based | Private medical center for children in the greater Helsinki area | 18 months | Both caregivers and physicians considered that the concerns and questions of caregivers of children were well handled in a external consultation service Web-based chat |
| Nadia, B [37] | 2022 | Has Telemedicine come to Fruition? Parents’ and Physicians’ Perceptions and Preferences Regarding Telemedicine | Quantitative | To evaluate both patient and physician perceptions, preferences, and acceptability regarding the use of the different modalities of telemedicine for various health problems | Parents of children (n=222), Pediatrician (n=45) | Ambulatory | University of Geneva | 12 months | Parents preferred the telephone for simple medical advice, discussion of parameters, acute or chronic problems, and psychological support |
| Sarabu, C [28] | 2021 | The Value of OpenNotes for Pediatric Patients, Their Families and Impact on the Patient-Physician Relationship | Quantitative | To better understand how pediatric patients and families perceived OpenNotes | Parents of children <12 years (n=159) | Ambulatory | Stanford Children's Health | 5 month | Majority of patients and families understood their notes; they largely found them accurate; most did not contact their clinicians after reading a note; and the majority found that reading their notes improved trust with their clinician |
| Weems, M [38] | 2016 | Electronic communication preferences among mothers in the neonatal intensive care unit | Quantitative | To assess the use of online and mobile communication platforms among mothers of NICU infants and their interest and preferences in receiving electronic clinical updates during the NICU stay using available technology | Mothers of infants admitted to the NICU (n=217) | NICU (2) | Regional One Health, Le Bonheur Children's Hospital | 3 months | Mobile technology is widely used by this population of NICU mothers and could be a powerful tool to help mothers cope and care for infants during and after admission to the NICU |
| Adams, S [18] | 2021 | Perspectives on team communication challenges in caring for children with medical complexity | Qualitative | Explore communication challenges and solutions/recommendations from multiple perspectives (parents of children with medical complexity, health care providers, teachers) with a goal of informing patient care | Primary caregivers of a medically complex child (n=17), Health care provider (n=11), Teachers (n=5) | Community | Hospital for Sick Children, Credit Valley Hospital, and Royal Victoria Regional Health Centre | 18 months | There were three types of communication challenges described by parents, HCPs, and teachers: 1) Organizational policy and antiquated technology system barriers, 2) Inadequate access to health information and 3) Lack of partnership in clinical decision making |
| Britto, M [24] | 2013 | Parents' Perceptions of a Patient Portal for Managing Their Child's Chronic Illness | Qualitative | To examine parents' perceptions of the benefits and/or drawbacks of a patient portal for managing their child's chronic illness | Parents of child aged <18 years diagnosed with chronic illness (n=24) | Ambulatory | Cincinnati Children's Hospital | Not disclosed | Reported perceived portal benefits include removing barriers to communication, reducing hassles, maximizing convenience, providing a sense of control and independence, reducing anxiety, and providing reassurance |
| Horsky, J [19] | 2014 | Coordination of Care for Complex Pediatric Patients: Perspectives from Providers and Parents | Qualitative | To gather and analyze insights from clinicians, coordinators, and parents of children with multiple chronic conditions to formulate a preliminary model of care coordination intended to inform the design of electronic support tools | Parents of children with multiple chronic problems (n=16), Medical care providers (n=6) | Ambulatory; Community | Two institutions affiliated with a large urban healthcare delivery network | 5 hours | The core themes about barriers to effective care coordination that emerged from the interviews showed that lack of integration and system interoperability within and primarily across institutions and professions adds considerable effort to the work of most clinicians, providers of services, and parents |
| Kelly, M [22] | 2019 | Parent Perceptions of Real-time Access to Their Hospitalized Child's Medical Records Using an Inpatient Portal: A Qualitative Study | Qualitative | To identify why parents used the portal, their suggestions for improvement, and their perspectives of new features being considered by the hospital | Parents of hospitalized children < 12 years (n=14) | Inpatient | Tertiary care children's hospital in the Midwest | Not disclosed | Providing parents with real-time clinical information during their child's hospitalization using an inpatient portal may enhance their ability to engage in caregiving tasks critical to ensuring inpatient care quality and safety |
| Kelly, M [27] | 2021 | Stakeholder Perspectives in Anticipation of Sharing Physicians' Notes With Parents of Hospitalized Children | Qualitative | Elicit stakeholder perspectives on the anticipated benefits and challenges of sharing hospital physicians' admission and daily progress notes with parents at the bedside during their child's hospitalization and identify strategies to aid implementation of inpatient note sharing | Parents of hospitalized children (n=8), Nurses (n=8), Residents (n=5), Hospitalists (n=7), Administrators (n=6) | Inpatient | Tertiary children's hospital in the Midwest | 2 months | Parent and healthcare team stakeholders anticipate benefits and challenges of sharing inpatient notes with parents during their child's hospitalization and suggest practical ways that hospitals can implement note sharing to support positive outcomes and mitigate negative consequences |
| Kelly, M [51] | 2023 | Parent Perceptions of Real-time Access to Their Hospitalized Child's Medical Records Using an Inpatient Portal: A Qualitative Study | Qualitative | To identify why parents used an inpatient portal application on a tablet computer during their child's hospitalization and identify their perspectives of ways to optimize the technology | Parents of hospitalized children < 12 years (n=14) | Inpatient | Tertiary care children's hospital in the Midwest | Not disclosed | Providing parents with real-time clinical information during their child's hospitalization using an inpatient portal may enhance their ability to engage in caregiving tasks critical to ensuring inpatient care quality and safety |
| Smith, C [52] | 2022 | In Anticipation of Sharing Pediatric Inpatient Notes: Focus Group Study With Stakeholders | Qualitative | To investigate the anticipated impact of increasing the flow of electronic health record information, specifically physicians’ daily inpatient progress notes, via a patient portal to parents during their child’s acute hospital stay—an understudied population and an understudied setting | Parents of children <12 years (n=8), Hospital administrators (n=6), Hospitalist physicians (n=7), Resident physicians (n=5), Nurses (n=8) | Inpatient | Midwest academic children's hospital | 2 months | All focus groups identified many potential benefits of inpatient Open Notes inlcuding the enhanced sharing of information between the health care team and absent family members; increasing information for parents to review, thus adding to their knowledge base; providing parents with a sense of structure, enabling them to plan and organize; improving quality assurance for the health care system by involving parents as viewers, commenters, and potential correctors of the record; and illuminating the clinical communication process itself, thus educating and reassuring parents about the care process. |
| Aldekhyyel, R [53] | 2018 | Using a Bedside Interactive Technology to Solicit and Record Pediatric Pain Reassessments: Parent and Nursing Perspectives on a Novel Workflow | Mixed-Methods | Study the perspectives of both parents and nurses towards using an interactive patient care tool in the management of pain at our children's hospital | Parent of hospitalized child (n=30), Nurses (n=59) | Inpatient | University of Minnesota Masonic Children's Hospital | 6 months | Cohesive agreement among parents and nurses on the perceived usefulness of the pain management tool |
| King, G [54] | 2017 | Connecting Families to Their Health Record and Care Team: The Use, Utility, and Impact of a Client/Family Health Portal at a Children's Rehabilitation Hospital | Mixed-Methods | To examine the use, utility, and impact of the connect2care portal from the beginning of portal introduction until the end of data collection | Caregivers of children (n=38); Providers (n=9) | Inpatient; Ambulatory; Community | Holland Bloorview Kids Rehabilitation Hospital | 14 months | There was a moderate degree of perceived usefulness of and satisfaction with the EHR and e-messaging features, and evidence that the portal was perceived to provide useful access to the clinical record |
| Parpia, C [30] | 2021 | Evaluation of a Secure Messaging System in the Care of Children With Medical Complexity: Mixed Methods Study | Mixed-Methods | To (1) evaluate the use of a secure messaging system, (2) examine and compare the content of messages to email and phone calls, and (3) explore PCs' and CTMs' perceptions and experiences using secure messaging as a method of communication | Parental caregivers of children with medical complexity <18 years (n=36), Nurse practitioners (n=7), Other Hospital and community-based healthcare providers (n=59) | Community | The Hospital for Sick Children, Credit Valley Hospital, and Royal Victoria Regional Health Centre | Not disclosed | Secure messaging was highly used, allowed for diverse topics of conversation, and enhanced the PC-CTM relationship |
| Schiller, J [33] | 2013 | What parents want from emails with their pediatrician: Implications for teaching communication skills | Mixed-Methods | To elicit patient preferences about email communication to inform training. To elicit parents' perspectives on physician-parent email communication and compared parent and faculty assessments of medical students' emails | Parents of children (n=19) | Ambulatory | Not disclosed | 3 months | Parents place value on medical students' abilities to communicate clearly and convey respect and empathy in email |
| Weatherly, J [20] | 2019 | Challenges with Patient Adoption of Automated Integration of Blood Glucose Meter Data in the Electronic Health Record | Mixed-Methods | To understand how AIS would impact patient-provider communication | Patients with type 1 diabetes age 5-20 years or their parents (n=28) | Ambulatory | Stanford Children's Health | 6 month | Although integration is technically possible, the patient experience was cumbersome and resulted in low adherence to the technology |
